# Specific Polyunsaturated Fatty Acids Can Modulate *in vitro* Human moDC2s and Subsequent Th2 Cytokine Release

**DOI:** 10.3389/fimmu.2020.00748

**Published:** 2020-05-04

**Authors:** Tamara Hoppenbrouwers, Vincenzo Fogliano, Johan Garssen, Nicoletta Pellegrini, Linette E. M. Willemsen, Harry J. Wichers

**Affiliations:** ^1^Food Quality and Design, Wageningen University & Research, Wageningen, Netherlands; ^2^Division of Pharmacology, Department of Pharmaceutical Sciences, Faculty of Science, Utrecht University, Utrecht, Netherlands; ^3^Department of Immunology, Nutricia Research BV, Utrecht, Netherlands; ^4^Food and Biobased Research, Wageningen University & Research, Wageningen, Netherlands

**Keywords:** DC2, Th2, DC2–T-cell model, polyunsaturated fatty acid, allergy, docosahexaenoic acid

## Abstract

Allergy is becoming a rapidly increasing problem worldwide, and *in vitro* models are frequently used to study the mechanisms behind the different types of allergic response. The dendritic cell (DC)–T-cell model can be used to study sensitization. However, lipopolysaccharide (LPS) is often used to maturate the DCs, but it gives rise to a DC1 phenotype, whereas Th2-driven inflammatory diseases such as allergy are characterized by the involvement of the DC2 phenotype. Our aim was to create a DC2–T-cell human model (human moDC2s) to study *in vitro* sensitization and validate the model using polyunsaturated fatty acids (PUFAs) that were previously shown to have immunomodulatory properties. We found that the generated DC2s expressed OX40L and drove naive T-cells into IL-13 production of CD4^+^ effector T-cells. In line with *in vivo* findings, *n*−3 long-chain (LC)PUFA docosahexaenoic acid (DHA) effectively decreased the DC2's surface expression of OX40L, as well as the IL-12p40 and IL-23 cytokine production by DC2s and subsequently lowered IL-13 production by DC2-induced effector T-cells. Similar cytokine production effects were found with eicosapentaenoic acid (EPA) and arachidonic acid (AA), whereas linoleic acid (LA) increased OX40L surface expression and subsequent T-cell-derived IL-13/IFNγ ratios, suggesting an increased risk of allergy development. Altogether, these data show that human moDC2s are able to induce Th2-type IL-13 secretion by T-cell differentiated in the presence of these DC2s and that this model can be differentially modulated by PUFAs. These results are in line with previous *in vivo* studies using PUFAs, indicating that this model may be of use to predict *in vivo* outcomes.

## Introduction

Th2-driven inflammatory diseases, such as allergic asthma, allergic rhinitis, and food allergy, are rapidly increasing worldwide. According to the European Academy of Allergy and Clinical Immunology (EAACI), in Europe, already 150 million people suffer from some type of allergy, and around 50% of all Europeans will be allergic in 2025, with no age, social, or geographical distinctions (1). Therefore, new strategies in allergy prevention and treatment are very much needed.

Currently, most allergy studies are performed using preclinical animal (mouse) models (2, 3). On the one hand, this offers a preclinical model in which all essential immune compartments to elicit an allergic reaction are operational. On the other hand, the possibility to translate findings to the human situation is not always straightforward. Often, biopsies from allergic patients or animal's lung or gut are used (4); however, obtaining these specimens can be limiting the research opportunities. *In vitro* allergy models often consist of single-cell models, such as mast cells (MCs) or basophils (5). MCs or basophils represent cells of the allergic effector phase because their activation results in the acute allergic symptoms, meaning that in these models, allergy treatment can be studied *in vitro* rather than allergy prevention. To study allergy prevention, the sensitization phase is of great importance. One of the most important cellular interactions in the development of allergic sensitization is the dendritic cell (DC)–T-cell interaction (6, 7). DCs are important in allergen uptake at the mucosal tissue and consequently major histocompatibility complex (MHC) type II-associated presentation of the allergic epitopes to the specific T-cell receptors. Depending on the phenotype of these DCs, which can be modified by their cellular and molecular microenvironment, they can either instruct tolerance or drive effector T-cell responses such as Th2 cells that contribute to the allergic sensitization cascade. Hence, the DCs and naive T-cell interaction and consequent development of Th2 cells provide an interesting model to preselect various bioactive preparations aimed at allergy management. Monocyte-derived DCs and allogeneic naive T-cell interaction studies are commonly performed *in vitro* to investigate antigen presentation and T-cell proliferation, differentiation, and related cytokine response. To obtain mature DCs, immature monocyte-derived DC (iDCs) are often incubated with lipopolysaccharide (LPS), which in most cases leads to DCs that drive Th1 differentiation (8). Protocols for the generation of DC2s, which are described to be involved in Th2 differentiation, have been published, although these are not many and, especially, not frequently used to study the capacity of bioactive components for sensitization prevention (9). The protocol described by Kalinski et al. (9) uses *Staphylococcus aureus* enterotoxin B (SEB) to induce Th2 cell differentiation, which has been described to skew Th2 differentiation by itself, without needing the DC2 phenotype (10). Therefore, we will further explore the potential of a DC2–T-cell model without the use of SEB.

Many allergy prevention studies have been performed using polyunsaturated fatty acids (PUFAs) (11). The most prominently studied PUFA groups are omega-3 (*n*−3) and omega-6 (*n*−6) of which *n*−3 α-linolenic acid (ALA) and *n*−6 linoleic acid (LA) are essential in the human diet, and these can be mainly obtained from vegetable oils, seeds, and nuts. They can be elongated and desaturated *in vivo* to obtain the more bioactive long-chain (LC)PUFA. Alternatively, these can be directly obtained *via* the diet. *In vivo*, fish oil rich in *n*−3 LCPUFAs EPA and docosahexaenoic acid (DHA) has been shown to prevent allergic responses in allergic mouse (12, 13) and guinea pig (14) models. *In vitro* and *ex vivo, n*−3 PUFAs EPA and DHA inhibited costimulatory molecules CD80 and CD86 on DCs and reduced DC inflammatory cytokine production (15, 16), T-cell proliferation (17, 18), and response to DCs (19). Furthermore, *n*−6 PUFA AA has mostly been studied in the effector phase. *n*−6 PUFAs were shown to enhance MC TNFα production and IgE-mediated degranulation (20, 21), which was also observed *in vivo* (22). Of note, the majority of the DC–T-cell studies published were performed in LPS-stimulated DCs. Therefore, the current study aimed to establish an *in vitro* DC2–T-cell model to more effectively investigate the sensitization phase, validated by investigating the immunomodulatory effects of PUFAs, including *n*−6 PUFAs LA and AA as well as *n*−3 PUFAs ALA, EPA, and DHA.

## Materials and Methods

### Monocyte and T-Cell Isolation

Primary human monocytes were isolated from buffy coats from healthy donors (Sanquin, Nijmegen, The Netherlands). Before sample collection, a written consent was obtained. Buffy coats were diluted 1:1 with phosphate-buffered saline (PBS) + 2% fetal bovine serum (FBS) (HyClone™ Fetal Bovine Serum, Fisher Scientific, Loughborough, UK) and loaded onto Greiner Bio-One™ LeucoSEP™ Polypropylene Tubes to obtain peripheral blood mononuclear cells (PBMCs). After being washed, the cells were diluted in MACS buffer, and a CD14 microbead kit was used to isolate the monocytes according to the manufacturer's protocol (Miltenyi Biotec, Leiden, The Netherlands). Naive CD4^+^ T-cells were isolated from the flow through using a negative selection. A naive CD4^+^ T Cell Isolation Kit, containing a cocktail of biotinylated CD45RO, CD8, CD14, CD15, CD16, CD19, CD25, CD34, CD36, CD56, CD123, anti-TCRγ/δ, anti-HLA-DR, and CD235a (glycophorin A) antibodies (Miltenyi Biotec, Leiden, The Netherlands), was used. All cells were frozen in 1:1 FBS and FBS with 20% dimethyl sulfoxide (DMSO) and stored at −80°C until further use.

### Dendritic Cell Differentiation and Polyunsaturated Fatty Acid Treatment

iDCs were obtained by culturing primary monocytes for 6 days in a 24-well plate in Iscove's Modified Dulbecco's Medium (IMDM, Gibco Thermo Fisher, Landsmeer, The Netherlands) with 10% FBS and 1% p/s in the presence of 30 ng/ml of IL-4 (Sigma Aldrich, Zwijndrecht, The Netherlands) and 50 ng/ml of granulocyte-macrophage colony-stimulating factor (GM-CSF) (R&D Systems, Minneapolis, USA). To differentiate into DC2, a maturation mix containing 50 ng/ml of TNFα (Miltenyi Biotec, Leiden, The Netherlands), 25 ng/ml of IL-1β (Merck Millipore, Amsterdam, The Netherlands), 100 U/ml of IL-6 (Miltenyi Biotec, Leiden, The Netherlands), and 1 μg/ml of PGE_2_ (Sigma Aldrich, Zwijndrecht, The Netherlands) was used, as described previously (9). As a control, DCs were also matured using 100 ng/ml of LPS (γ-irradiated LPSs from *Escherichia coli* O111:B4, Sigma Aldrich, Zwijndrecht, The Netherlands). For PUFA treatment, iDCs were incubated with 75 μM of vitamin C (23) and 100 μM (24) of LA, ALA, AA, DHA, or EPA (all from Sigma Aldrich, Zwijndrecht, The Netherlands) and 20 μM of vitamin E (23), which was resuspended in the serum before adding to the culture medium and cells. To control for the effect of vitamins C and E, the measurements of the NT DC2s were also compared with DC2s treated with only vitamins C and E. After 48 h, the iDCs were incubated for another 48 h with the DC2 maturation mix. Subsequently, the maturated moDCs were harvested and stained for flow cytometry, and the supernatant was stored at −20°C for further analysis.

### Dendritic Cell–T-Cell Co-culture

DC2s treated with PUFAs were cultured in a flat-bottom 96-well plate and obtained as described above. After maturation, cells were harvested and replated into a round-bottom 96-well plate. Naive CD4^+^ T-cells were added 10:1 with 5 ng/ml of IL-2 (Miltenyi Biotec, Leiden, The Netherlands) and 150 ng/ml of anti-CD3 antibody (Clone CLB-T3/4.E, 1XE, Sanquin, Amsterdam, The Netherlands). After 5 days, the cells were harvested and stained for flow cytometry, and the supernatant was stored at −20°C for further analysis.

### Flow Cytometry

To analyze surface marker expression of the DCs, cells were stained with anti-CD14 (PE-eFluor 610, Thermo Fisher, Landsmeer, The Netherlands), anti-HLA-DR (APC-eFluor 780, Thermo Fisher, Landsmeer, The Netherlands), anti-CD11c (PE-Cy5.5, Thermo Fisher, Landsmeer, The Netherlands), anti-CD80 (PE-Cy7, BioLegend, Koblenz, Germany), anti-CD83 [fluorescein isothiocyanate (FITC), Thermo Fisher, Landsmeer, The Netherlands], anti-CD86 [antigen-presenting cell (APC), BioLegend, Koblenz, Germany], and anti-OX40L [phycoerythrin (PE), BioLegend, Koblenz, Germany] and analyzed using a CytoFLEX flow cytometer (Beckman Coulter, Woerden, The Netherlands). Cell viability in DCs was determined using 7AAD dye (BD Pharmingen, San Jose, CA).

### Enzyme-Linked Immunosorbent Assay

In the supernatant of the matured moDCs, IL-12/IL-23 (p40) was measured according to the manufacturer's protocol (BioLegend, Koblenz, Germany). As such, IL-13, IFNγ, and IL-10 (from BioLegend, Koblenz, Germany) were determined in the supernatant of the DC–T-cell co-cultures. All samples were measured using a Tecan Infinite 200PRO (Tecan, Männedorf, Switzerland).

### LEGENDplex

To further investigate cytokine production of the DC2s, LEGENDplex a panel for Human Macrophage/Microglia, was used, according to the manufacturer's protocol (BioLegend, Koblenz, Germany). Cytokines in the Human Macrophage/Microglia panel included IL-12p70, TNFα, IL-6, IL-4, IL-10, IL-1β, arginase, TARC, IL-1RA, IL-12p40, IL-23, IFNγ, and IP-10. All samples were measured using a CytoFLEX flow cytometer (Beckman Coulter, Woerden, The Netherlands), and data were analyzed using the BioLegend LEGENDplex cloud-based software.

### Cytokine Ratio Calculations

To analyze the balance of the different cytokines produced as a reflection of T-cell subsets, the ratios were calculated of the normalized data. For each individual donor, the T-cell-derived production of IL-13, IFNγ, or IL-10 of each PUFA treatment was divided by the intrinsic production of the T-cells exposed to untreated DC2s. The production of the control condition was set to 1 for each cytokine. Then, IL-13/IFNγ ratios were calculated by dividing the means of the IL-13 production by the means of the IFNγ production for each individual donor. Similarly, we calculated the IL-13/IL-10 and IFNγIL-10 ratio.

### Statistics

All experiments were repeated at least three times independently with different donor cells (*n* = independent biological replicates). Surface marker expression of iDCs, DC1s, and DC2s were *n* = 3. Surface marker expression of DC2s + PUFAs was *n* = 5. All LEGENDplex and ELISA experiments were *n* = 5, except for IL-13, which was *n* = 7. Statistical analyses were carried out using IBM SPSS Statistics 23. All parameters are presented as means ± SEM. Outliers were calculated by determining Q1 and Q3 of the dataset, thereby defining the interquartile range (IQR). Subsequently, the L-bound and U-bound were calculated by subtracting (1.5 ^*^ IQR) from the Q1 (L-bound) or adding (1.5 ^*^ IQR) to the Q3 (U-bound). Outliers were defined as values < L-bound or >U-bound and removed from the dataset. Outliers were *n* = 5 in total, randomly divided over the dataset and groups. A mixed linear model, based on a repeated measures ANOVA, with a Bonferroni *post-hoc* to correct for multiple comparisons was used to assess the parameters for significance if *p* < 0.05 (*p* < 0.1 is considered as trend), where different treatments on the same donor cells within one experiment were considered as paired data. Within each experiment, Bonferroni *post-hoc* tests were performed on selected groups, and all groups were only compared with the non-treated (NT) DC2 control. Graphs were plotted using GraphPad Prism 8.

## Results

### DC2 Surface Marker Expression

**Figure 1:**
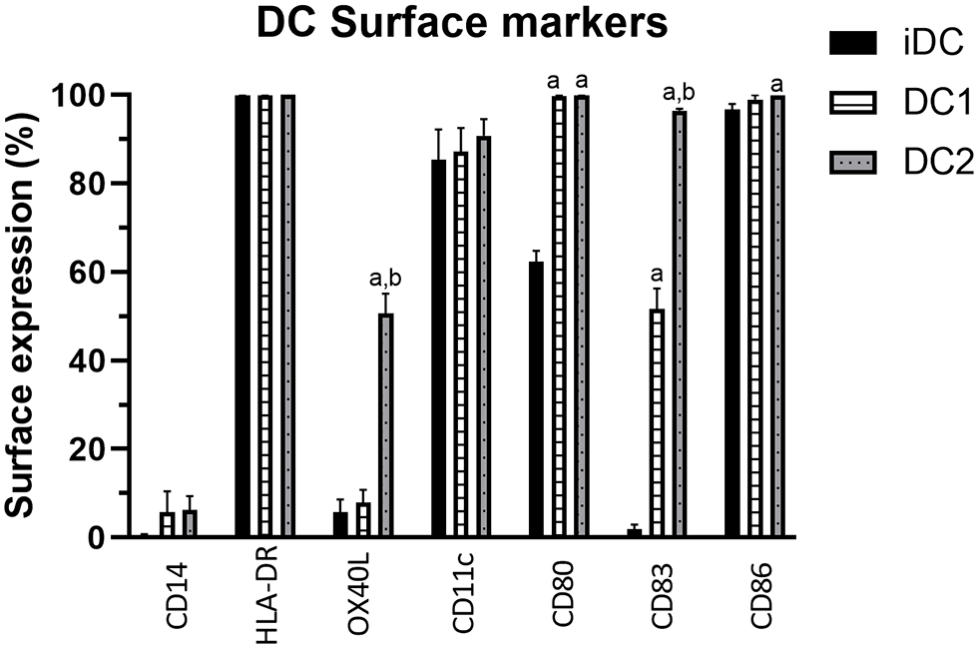
Percentage of DC surface marker expression on iDCs, DC1s, and DC2s as measured by flow cytometry. Percentages indicate the frequency of cells showing surface marker expression of total cells measured. Means ± SEM. *N* = 3 independent donors. **(A)** Significantly different from iDCs. **(B)** Significantly different from DC1s. DC, dendritic cell; iDCs, immature monocyte-derived DCs.

DC2 cells were characterized by comparing their surface marker expression to iDCs and DC1s, where OX40L was used as a marker for DC2s. Immature DCs did not express much CD14, which is lost upon transition from monocyte to DCs. iDCs did already express DC surface markers HLA-DR, CD11c, and CD86, and to some extent CD80 and CD83 (Figure 1). CD86 was significantly higher in DC2s compared with iDCs (*p* = 0.04). CD80 and CD83 were significantly higher in DC1s (*p* < 0.001 and *p* = 0.01, respectively) and DC2s (*p* < 0.001 and *p* = 0.003, respectively) compared with iDCs. CD83 was significantly higher in DC2s compared with DC1s (*p* < 0.001), indicating a different phenotype of maturation. Finally, OX40L was significantly higher in DC2s compared with iDCs and DC1s (50.8 ± 4.4%, 5.7 ± 3.0%, and 7.9 ± 2.9%, *p* < 0.001, respectively), showing that the used cytokine mixture was capable of phenotypically inducing DC2 maturation as indicated by surface marker expression.

### DC2 Cytokine Production

To further investigate the differences between DC2s and DC1s, 13 different cytokines were measured in the supernatant after 48 h of maturation using the Macrophage/Microglia LEGENDplex. DC2s produce significantly more TNFα, IL-1β, and IL-23 than do DC1s (Table 1). Furthermore, DC1s produce significantly more IP-10, although in very low amounts. All other measured cytokines were similarly produced. Of note, IL-12p70 and IFNγ were undetectable in both DC2s and DC1s.

**Table 1:** Cytokines produced by DC2s vs. DC1s as measured by LEGENDplex analyses.

**Cytokine**	**DC2**	**DC1**	***p*-value**
IL-12p70	n.d.	n.d.	-
TNFα	13.6 ± 1.9	3.3 ± 1.1	0.004^**^
IL-6	7.4 ± 2.0	17.6 ± 8.0	0.27
IL-4	0.2 ± 0.1	0.4 ± 0.2	0.52
IL-10 (pg/ml)	2.0 ± 0.9	70.0 ± 50.0	0.25
IL-1β	9.1 ± 1.6	0.003 ± 0.0007	0.005^**^
Arginase	74.1 ± 5.0	79.8 ± 9.9	0.67
TARC	2.3 ± 0.1	2.1 ± 0.2	0.23
IL-1RA	12.6 ± 2.4	16.1 ± 1.1	0.31
IL-12p40	6.2 ± 1.7	6.8 ± 3.4	0.76
IL-23 (pg/ml)	30.0 ± 9.0	5.0 ± 3.0	0.04^*^
IFNγ	n.d.	n.d.	-
IP-10 (pg/ml)	2.0 ± 1.0	30.0 ± 7.0	0.03^*^

*Values are presented in ng/ml, unless stated differently. Means ± SEM. n.d. indicates not detectable. N = 5 independent donors. DC, dendritic cell*.

* *p < 0.05*.

** *p < 0.01*.

*** *p < 0.001*.

### Polyunsaturated Fatty Acids Can Modulate Surface Marker Expression in DC2s

*n*−3 (ALA, EPA, and DHA) and *n*−6 (LA and AA) PUFAs, which have been previously described to affect surface expression on LPS maturated DCs (15, 16), were selected to study modulation of surface marker expression. As vitamins C and E were to be added to avoid lipid oxidation of the PUFAs, a medium containing only these vitamins was taken along as the proper control. Viability staining (7AAD) indicated that all cells were viable after PUFA treatment (no toxicity and cell death as compared with that of the control, data not shown). The surface expression values of the measured markers can be found in Table 2.

**Table 2:** Modulation of DC2 surface expression markers by PUFAs.

	**CD14**	**HLA-DR**	**OX40L**	**CD11c**	**CD80**	**CD83**	**CD86**
NT	4.4 ± 1.5	98.0 ± 0.8	49.0 ± 8.2	82.3 ± 7.2	88.1 ± 4.2	83.8 ± 4.1	93.5 ± 6.0
Vitamin	3.2 ± 0.8	96.7 ± 1.4	36.5 ± 13.1	84.7 ± 4.8	88.3 ± 2.9	83.5 ± 4.9	91.8 ± 4.9
*p*-value	1.00	1.00	0.92	1.00	1.00	1.00	1.00
*n*−6							
LA	5.3 ± 4.4	99.0 ± 0.4	80.7 ± 9.5	77.4 ± 10.9	72.3 ± 3.5	79.3 ± 2.6	99.0 ± 0.8
*p*-value	1.00	1.00	0.06	1.00	0.67	1.00	1.00
AA	5.5 ± 2.7	93.2 ± 2.6	11.8 ± 1.6	79.5 ± 5.4	62.2 ± 4.8	58.2 ± 7.5	82.3 ± 10.5
*p*-value	1.00	1.00	0.08	1.00	0.02^*^	0.04^*^	1.00
*n*−3							
ALA	1.1 ± 0.4	99.2 ± 0.2	72.1 ± 12.6	69.3 ± 9.5	69.1 ± 10.5	75.5 ± 7.1	99.3 ± 0.1
*p*-value	0.68	1.00	0.71	1.00	0.37	1.00	1.00
EPA	2.7 ± 0.6	93.3 ± 5.3	23.2 ± 7.6	86.0 ± 4.4	77.3 ± 9.7	76.5 ± 7.8	89.8 ± 9.3
*p*-value	1.00	1.00	0.32	1.00	0.76	1.00	1.00
DHA	1.3 ± 0.5	93.0 ± 4.5	10.9 ± 2.3	72.9 ± 8.0	75.0 ± 4.7	67.3 ± 7.4	90.8 ± 7.8
*p*-value	0.71	1.00	0.05^*^	1.00	0.54	0.37	1.00

*Values are % as measured by flow cytometry. Means ± SEM. N = 5 independent donors*.

*DC, dendritic cell; NT, non-treated DC2; vitamin, vitamin-exposed DC2 control; PUFA, polyunsaturated fatty acid*.

* *p < 0.05*.

**Figure 2:**
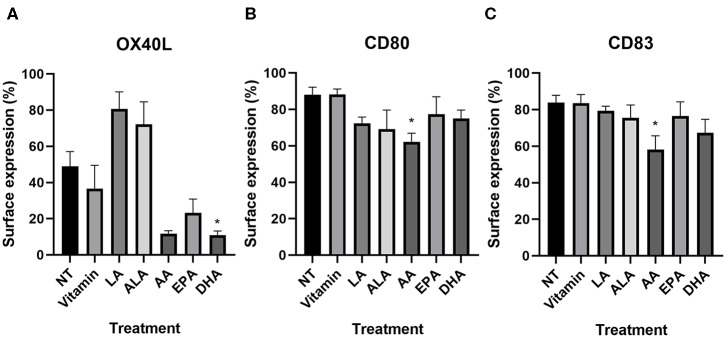
Percentage of surface expression of OX40L **(A)**, CD80 **(B)**, and CD83 **(C)** in DC2s treated with PUFAs as measured by flow cytometry. Means ± SEM. All experiments are *n* = 5 independent donors. *p < 0.05. DC, dendritic cell; PUFA, polyunsaturated fatty acid.

NT DC2s and vitamin-treated DC2s were similar in all different markers (Table 2). On all PUFA-treated DC2s, CD14 surface expression remained low and HLA-DR, CD11c, and CD86 expression remained high and did not differ significantly. OX40L was significantly lower in DHA-treated DC2s compared with the NT DC2s (*p* = 0.05) and showed a trend in AA-treated DC2s (*p* = 0.08) (Table 2 and Figure 2A). Furthermore, LA-treated DC2s showed an increased trend of OX40L (*p* = 0.06). Finally, AA significantly lowered surface expression of CD80 and CD83 than did NT DC2s (Figures 2B,C, *p* = 0.02 and *p* = 0.04, respectively).

### Polyunsaturated Fatty Acids Modulate Cytokine Expression of DC2s

Next, we investigated the effect of PUFAs on cytokine secretion. NT DC2s produced IL-12/IL-23 (p40), which was not affected by vitamin treatment (Supplementary Figure 1). Whereas, ALA did not have an effect, LA, AA, EPA, and DHA all significantly lowered IL-12/IL-23 (p40) production by DC2s compared with the NT [22.3 ± 5.1 vs. 12.0 ± 4.4 ng/ml, *p* = 0.02 (LA); 1.3 ± 0.5 ng/ml, *p* = 0.001 (AA); 4.1 ± 1.7 ng/ml, *p* < 0.0001 (EPA); and 6.0 ± 2.3 ng/ml, *p* = 0.005 (DHA), respectively].

**Figure 3:**
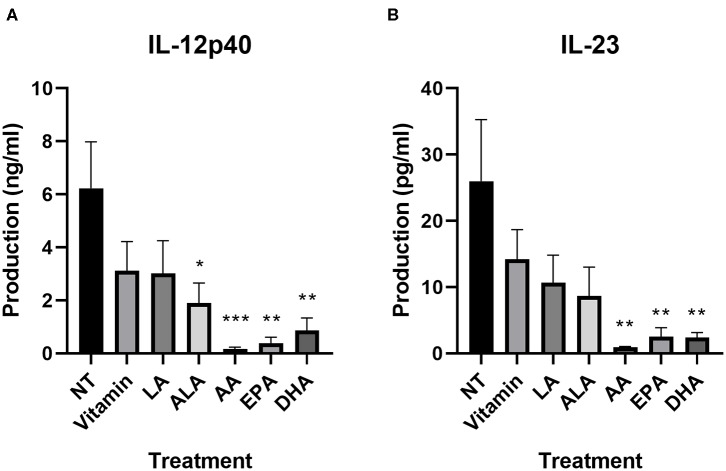
IL-12p40 **(A)** and IL-23 **(B)** production of DC2s after incubation with different PUFAs as measured using a LEGENDplex assay. All treatments were compared to the NT. Means ± SEM. All experiments are *n* = 5 independent donors. *p < 0.05; **p < 0.01; ***p < 0.001. DC, dendritic cell; PUFA, polyunsaturated fatty acid.

We further investigated cytokine production using a LEGENDplex kit. All values can be found in Supplementary Table 1. As also shown in the IL-12/IL-23 (p40) ELISA, all LCPUFAs affected the secretion of IL-12p40 and IL-23. Both cytokines were significantly lower in AA-treated (*p* = 0.001 and *p* = 0.004, respectively), EPA-treated (*p* = 0.002 and *p* = 0.008, respectively), and DHA-treated (*p* = 0.004 and *p* = 0.007, respectively) DC2s (Figure 3). ALA significantly lowered IL-12p40 and showed a trend for IL-23 (*p* = 0.03 and *p* = 0.08, respectively). All other DC2 produced mediators measured that remained unaffected by the PUFA treatment (Supplementary Table 1).

### DC2–T-Cell Activation

To validate the ability of the DC2s to drive Th2-type cytokine secretion by CD4^+^ T-cells as a reflection of Th2 development induced by the DC2, we analyzed the cytokine expression of the T-cells that were incubated with the DC2s for 5 days. Hallmark cytokines IFNγ (Th1), IL-13 (Th2), and regulatory IL-10 were compared between DC2 activated T-cells and T-cells activated by DC1s.

**Figure 4:**
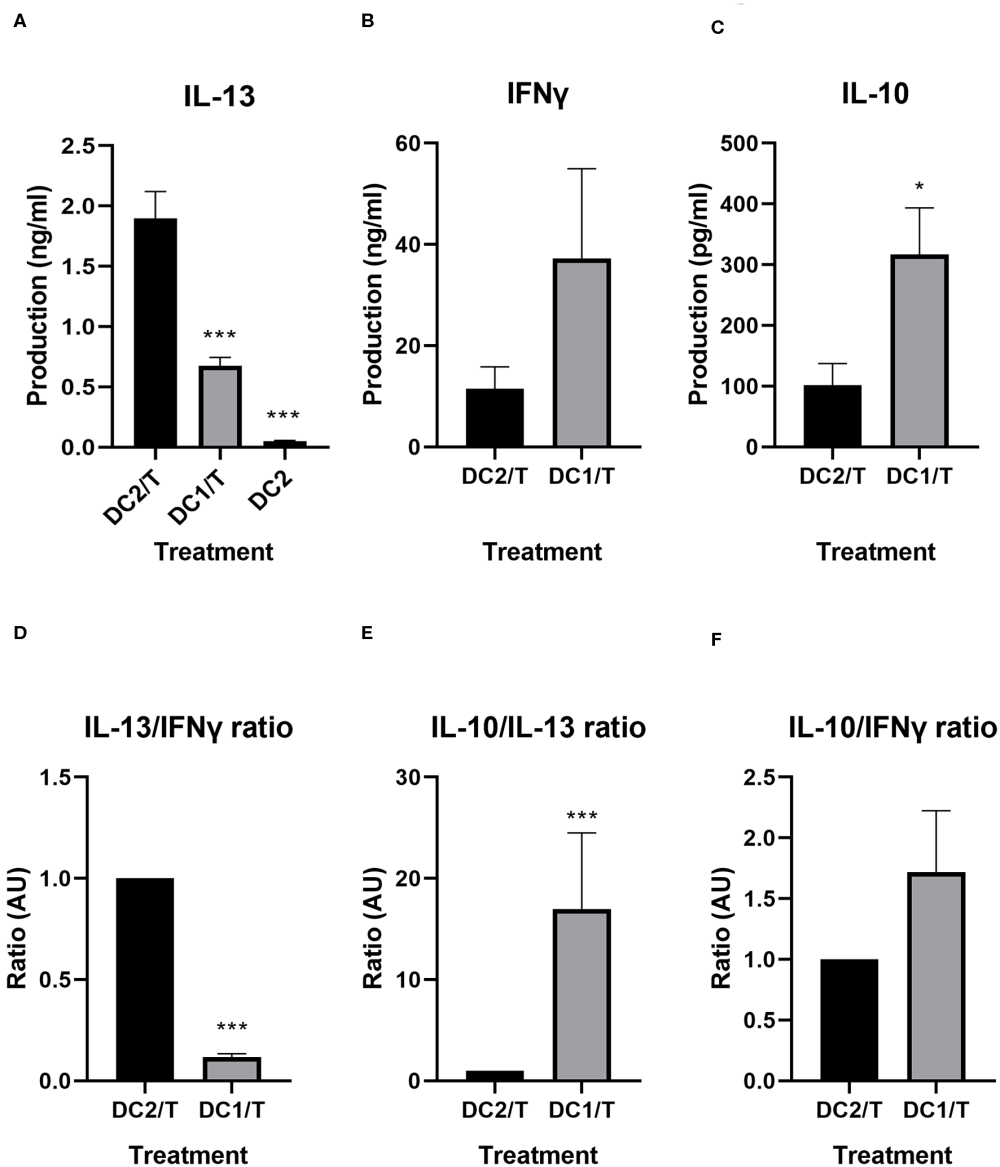
IL-13, IFNγ, and IL-10 production by T-cells incubated with DC2s or DC1s, as measured by ELISA **(A–C)** and ratios of IL-13/IFNγ, IL-10/IL-13, and IL-10/IFNγ compared between T-cells incubated with DC2s and T-cells incubated with DC1s **(D–F)**. All treatments were compared with those of the DC2/T. All IL-13 measurements **(A)** are *n* = 7 independent donors, except for DC2, which are *n* = 3 independent donors. All IFNγ and IL-10 measurements are *n* = 5. Means ± SEM. *p < 0.05; ***p < 0.001. DC, dendritic cell.

T-cells incubated with NT DC2s produced significantly more IL-13 than did T-cells incubated with DC1s (1.9 ± 0.2 vs. 0.7 ± 0.07 ng/ml, *p* = 0.001), thereby validating our model (Figure 4A). Of note, the DC2 themselves produced IL-13, but in very low quantities (50 ± 0.9 pg/ml). IFNγ production seemed to be, although not significant, slightly lower in T-cells incubated with NT DC2s compared with DC1s (Figure 4B). It must be noted that two of the five independent donors responded much less to LPS in IFNγ cytokine production, resulting in a large standard deviation. Finally, IL-10 was significantly lower in T-cells incubated with DC2s compared with T-cells incubated with DC1s (*p* = 0.02) (Figure 4C).

We calculated the ratios of hallmark cytokines IFNγ, IL-10, and IL-13 for the various treatments (Figures 4D–F). As expected, T-cells incubated with DC1s produce significantly less IL-13 over IFNγ (*p* < 0.001) than do T-cells incubated with untreated DC2s (Figure 4D). Furthermore, T-cells incubated with DC1s also produce significantly more IL-10 over IL-13 (*p* < 0.001) than do T-cells incubated with untreated DC2s (Figure 4E). Finally, production of IL-10 over IFNγ is similar between treatments (Figure 4F).

### Polyunsaturated Fatty Acid Modulate Th2-Type IL-13 Response Initiated by DC2s

**Figure 5:**
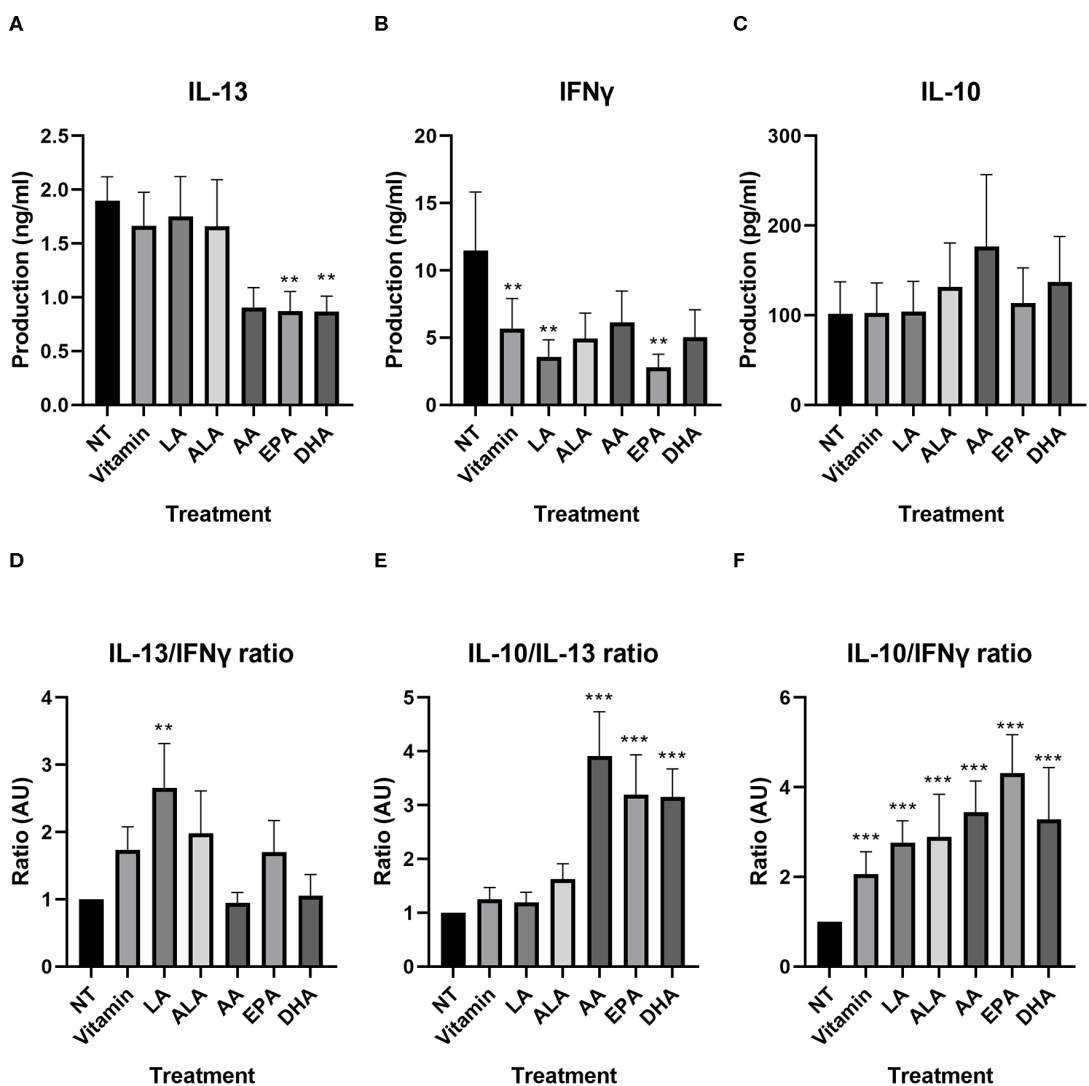
IL-13, IFNγ, and IL-10 production by T-cells incubated with DC2s treated with different PUFAs, as measured by ELISA **(A–C)** and ratios of IL-13, IFNγ, and IL-10 compared between T-cells incubated with DC2s that were treated with different PUFAs **(D–F)**. All treatments were compared with the NT (T-cells + DC2s). Means ± SEM. All IL-13 measurements are *n* = 7 independent donors. All IFNγ and IL-10 measurements are *n* = 5. **p < 0.01; ***p < 0.001. DC, dendritic cell; PUFA, polyunsaturated fatty acid.

We investigated whether PUFAs could inhibit production of these cytokines in our developed model. IL-13 production of T-cells incubated with NT DC2s and vitamin-treated DC2s was similar (Figure 5A). Furthermore, EPA and DHA significantly lowered IL-13 production of T-cells compared with T-cells incubated with NT DC2s [0.8 ± 0.2 ng/ml of EPA (*p* = 0.003) and 0.9 ± 0.1 ng/ml of DHA (*p* = 0.01)]. AA showed a trend [1.9 ± 0.2 vs. 0.9 ± 0.2 ng/ml of AA (*p* = 0.06)]. IFNγ production was significantly different between T-cells incubated with NT DC2s and vitamin-treated DC2s (11.5 ± 4. vs. 5.7 ± 2.2 ng/ml, *p* = 0.004), indicating an effect of vitamins alone (Figure 5B). This resulted in a significantly lowering effect of IFNγ by LA (*p* = 0.04) and EPA (*p* = 0.007), but not ALA, AA, and DHA. IL-10 production was similar between all treatments (Figure 5C).

We looked into the balance of different cytokines, similar to the comparison between T-cells incubated with DC2s and T-cells incubated with DC1s, as a reflection of the T-cell subjects over the different PUFA treatments (Figures 5D–F and Supplementary Table 2). The IL-13/IFNγ ratio was significantly higher when T-cells were incubated with DC2s treated with LA (*p* = 0.003), indicating that the effector T-cells develop toward a more Th2-type IL-13-directed response (Figure 5D). Other treatments gave similar IL-13/IFNγ ratios compared with those of the T-cells incubated with untreated DC2s. Interestingly, IL-10/IL-13 ratios were all significantly higher when T-cells were incubated with DC2s treated with AA, DHA, and EPA (all *p* < 0.001), indicating a higher production of regulatory cytokines (Figure 5F). There was no difference between IL-10/IL-13 ratios of T-cells incubated with untreated DC2s and T-cells incubated with DC2s treated with vitamin, LA, or ALA (Figure 5E). Moreover, all treatments gave significantly higher IL-10/IFNγ ratios than did T-cells incubated with untreated DC2s (all *p* < 0.001), indicating that vitamin can also skew the cytokine ratio from the hallmark Th1 cytokine IFNγ toward a more regulatory profile.

## Discussion

The data in this paper show that DC2s, generated from human primary monocytes, are able to induce the secretion of IL-13 by allogeneic Th cells as a reflection of Th2 cell development. This model can be used to analyze modulation of the DC2/T-cell interaction with PUFAs-modified DC. LCPUFA exposure of DCs during maturation with DC2 driving mediators resulted in a lower surface expression of different co-stimulatory markers for DCs (AA) or the DC2 marker OX40L (DHA). In addition, the cytokine production of both DC2s as well as IL-13 release by the developed T-cells and the IL-13/IFNγ ratio was reduced by specific LCPUFA while increasing the regulatory IL-10 over effector IL-13 and IFNγ balance. Together, this model can be used to study a variety of new potential allergy preventing components, which might aid in predicting *in vivo* outcomes.

DC–T-cell models often make use of DC1 cells that contribute to Th1 immunity and have been maturated using LPS. However, allergic sensitization is often characterized by the development of Th2-driving DC2. Hence, in order to study allergy prevention *in vitro*, maturation of the DCs toward a DC2 phenotype appears more relevant. Compared with the LPS maturated DCs, DC2 cells have a higher level of OX40L on the surface, as shown in our study and as described previously in monocyte-derived DC2 cells from allergic patients (25). This study by Gueguen et al. (25) extensively studied *in vitro* DC2s, maturated with IL-25, IL-33, LPS, PGE_2_, and thymic stromal lymphopoietin, compared with DC1s and DCregs. They observed that DC2s produced similar levels of IL-6, IL-8, IL-10, and TNFα and lower levels of IL-12p70 and IFNγ than did DC1s. Although IL-6 and IL-10 were, in line with these findings, similar; we found higher TNFα levels in DC2s compared with DC1s. In the current study, the IL-12p70 and IFNγ levels were below the detection limit in both DC1s and DC2s. However, the DC2s did produce detectable levels of IP-10 (CXCL10), which is closely linked to a Th1 driving signature, and these levels were low compared with those of DC1s. By contrast, the DC2s produced higher levels of IL-1β and IL-23 than did DC1s. Production of IL-1β and TNFα has also been found in IL-33-activated DCs that are associated with allergic airway inflammation (26). Moreover, our DC2s did produce low levels of IL-13 (32–63 pg/ml), in line with previous findings (25). Subsequently, the CD4^+^ T-cells incubated with DC2s produced significantly higher levels of IL-13 and tended to produce lower levels of IFNγ and IL-10 than did T-cells incubated with DC1s, as previously described (25). Finally, as expected, a higher IL-13/IFNγ ratio was observed in the supernatant of T-cells incubated with DC2s compared with that of T-cells incubated with DC1s. These findings indicate the expression of OX40L by the DC2 cells leading to the subsequent production of Th2 cytokine IL-13, which is a characteristic of DC2–T-cell interactions. The results of this study could be verified by comparing our findings to an assay using DCs and autologous T-cells derived from (food) allergic patients. In such a model, allergen-specific responses could be investigated for different types of allergies. As not all allergens are expected to be similarly modulated, a next step would be to analyze allergen-specific responses in our model. Altogether, the DC2–T-cell model might be a promising *in vitro* tool for screening new components for the prevention of Th2-driven inflammatory diseases. These types of models may be more relevant to predict the effects of bioactive components linked to an allergic setting than the DC–T-cell models that make use of LPS-induced activation.

The mechanism behind the effects of PUFA in DC2–T-cell activation remains unclear and needs further investigation. Th2 cells are commonly polarized by IL-4. *In vivo*, many IL-4 producing cells could be responsible for Th2 cell polarization. In an *in vivo* mouse model for ovalbumin (OVA)-induced food allergy, it has been shown that basophils could play an important role in IL-4-related polarization of Th2 cells in the sensitization phase of allergy (27), which has also been found *in vitro* (28). Furthermore, IL-4 production by MCs (29) and natural killer (NK) T-cells (30) has also been described to play a role in Th2 polarization and allergy. However, even though these cytokines were not added in the current set up and only low levels of IL-13 were measured in the DC supernatant, DC2s still were capable of instructing IL-13 release by the developed Th-cells, indicating that other factors beyond IL-4 and IL-13 also *in vivo* may be involved in development of Th2 driving DC.

The developed DC2-Th2 model was modulated by several PUFAs. In DC2s treated with LCPUFA DHA, the surface expression of OX40L was significantly lower than that of untreated DC2s and not significantly different from that of DC1s, indicating reduced DC2 development. This can also be observed in IL-12p40 and IL-23 cytokine production of the DHA-treated DC2s and reduced Th2 development as indicated by lower IL-13 production of the T-cells. Furthermore, LCPUFAs EPA (*n*−3) and AA (*n*−6) also significantly lower IL-12p40 and IL-23 cytokine production by DC2s similar to DHA and show a similar pattern in lowering T-cell-derived IL-13 secretion. In line with these findings, EPA and DHA have both been previously described to lower inflammatory cytokine production of DC1s (15, 16). However, inhibition of costimulatory molecules CD80 and CD86 (15, 16) was not observed by these *n*−3 LCPUFA in the DC2 model.

Surprisingly, AA was effective in lowering surface expression of CD80 and CD83 in addition to lowering the secretion of IL-12p40 and IL-23 by DC2s. Also in the subsequent DC2–T-cell assays, the Th2 response as indicated by IL-13 production was lowered by EPA and DHA and, although not significantly, by AA. Furthermore, the IL-13/IL-10 ratios were shifted more toward a regulatory IL-10 type of immune balance just like *n*−3 LCPUFA EPA and DHA. IL-10 is a suppressive cytokine that can be produced by several cell types, including regulatory T-cells. Indeed, regulatory T-cells were found to prevent the development of allergy in mice that were fed DHA-rich fish oil (12). Oral tolerance induction in mice was reported to be disrupted when an increasing dose of soy bean oil was provided, which contains no AA but LA. This increasing dose of soy bean oil was also found to increase the Th2/Th1 and Th2/Treg ratio, indicating skewing toward a pro-allergic phenotype, which may have facilitated allergic sensitization instead of oral tolerance induction. In the erythrocyte membranes of these mice, the LA levels increased, whereas AA remained unchanged (22). Indeed, in the current study, LA tended to increase OX40L in the DC2s, even though little difference was found in cytokine production between untreated DC2s and LA-treated DC2s. In line with this finding, we also observed a significantly higher IL-13/IFNγ ratio produced by T-cells incubated with DC2s treated with LA, indicating a shift toward a more Th2-type differentiation, as previously reported in allergic mice fed increasing concentration of LA-rich soybean oil (22). In conclusion, *in vivo* described allergy-reducing DHA was found to lower OX40L surface expression, and both EPA and DHA lowered IL-12p40 and IL-23 production by DC2s in association with less IL-13 production by T-cells. Also, EPA and DHA enhanced the secretion of regulatory type IL-10 secretion over Th2 and Th1 signature cytokines in the *in vitro* allogeneic DC2/T-cell model. In addition, *n*−6 PUFA LA tended to enhance Th2 driving OX40L expression on DC2 while enhancing the secretion of Th2-type IL-13 over Th1-type IFNγ, identifying LA as a possible factor contributing to allergic sensitization. By contrast, *n*−6 LCPUFA AA, similar to DHA, tended to lower OX40L expression and, in addition, was capable of lowering CD80 and CD83 costimulatory molecule expression on DC2 when added during maturation and tipping the balance toward a more regulatory-prone IL-10 response. This could indicate that like EPA and DHA, also AA may be able to lower the risk of allergic sensitization. Therefore, the developed allogeneic DC2/T-cell model might be useful for future testing of different anti-allergic bioactive components prior to testing them in *in vivo* animal or human studies. Furthermore, AA should be studied more extensively *in vivo* to further explore its potential in lowering allergic sensitization, maybe in combination with DHA in different ratios, as AA and DHA might compete in membrane incorporation.

As allergy is becoming a rapidly increasing worldwide problem, more tools are needed to investigate the allergic sensitization phase to be able to test agents that may prevent allergy from developing as the most important first step in allergy management. In this study, the use of the allogeneic DC2/T-cell model was shown as a promising model to investigate bioactive agents on several markers known to be involved in the allergic sensitization cascade (7). Not only *n*−3 LCPUFAs EPA and DHA successfully suppressed DC2 maturation and the consequent Th2-type IL-13 secretion by allogeneic T-cells. This effect was also shown by *n*−6 LCPUFA AA, unlike *n*−3 PUFA ALA and *n*−6 PUFA LA. This shows the discriminative capacity of this assay to predict the allergy preventive effect and to identify components that may be able to reduce allergic sensitization by targeting DC2 development. Future studies should further reveal the translational value of this model, for example, in *in vivo* preclinical models of allergic sensitization.

## Data Availability Statement

The datasets generated for this study are available on request to the corresponding author.

## Author Contributions

TH acquired and analyzed all data. TH and LW were responsible for the statistical analysis. All authors participated in the design and interpretation of the reported experiments and results, participated in drafting, and revising the manuscript.

## Conflict of Interest

JG was employed by Nutricia Research BV. The remaining authors declare that the research was conducted in the absence of any commercial or financial relationships that could be construed as a potential conflict of interest.
