# Supplementary material for: Specific Polyunsaturated Fatty Acids Can Modulate in vitro Human moDC2s and Subsequent Th2 Cytokine Release
Source: Front Immunol. 2020 May 4;11:748. doi: 10.3389/fimmu.2020.00748 (PMC7212991; doi:10.3389/fimmu.2020.00748)
Supplement: Supplementary file 1 [file Data_Sheet_1.docx]

**Supplementary data**


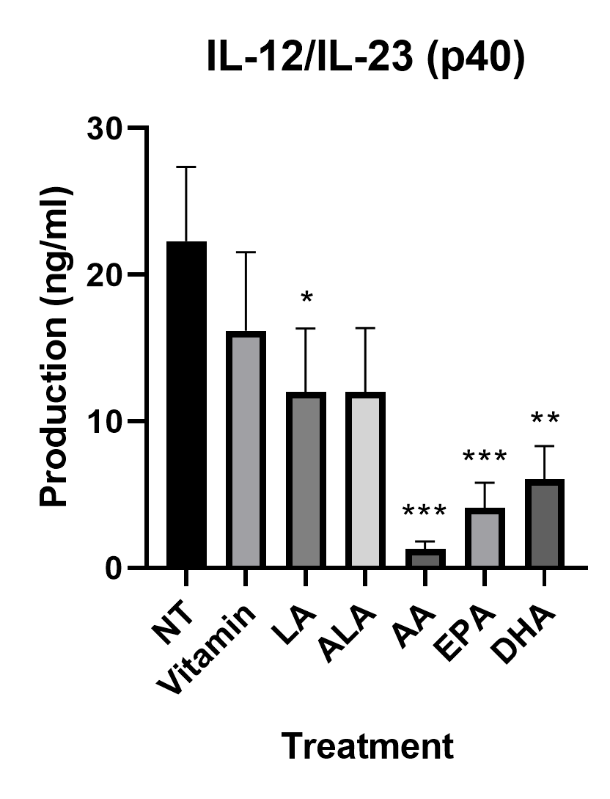


**Supplementary Figure 1:** IL-12/IL-23 (p40) production of DC2s after incubation with different PUFAs as measured by ELISA. All treatments were compared to the NT. Means ± SEM. All experiments are n=8 independent donors, except for LA and ALA, which are n=5 independent donors.
* p<0.05
** p<0.01
*** p<0.001

**Supplementary Table 1**:Cytokines produced by DC2s treated with different PUFAs as measured by Legendplex analyses. Values are presented in ng/ml. Means±SEM. N.d. indicates not detectable. N=5 independent donors.
* p<0.05
** p<0.01
*** p<0.001

|  | **TNFα** | **IL-6** | **IL-4** | **IL-10** | **IL-1β** | **Arginase** | **TARC** | **IL-1RA** | **IL-12p40** | **IL-23** | **IP-10** |
| --- | --- | --- | --- | --- | --- | --- | --- | --- | --- | --- | --- |
| **NT** | 13.6±1.9 | 7.4±2.0 | 0.2±0.09 | 0.002±0.001 | 9.1±1.6 | 74.2±5.0 | 2.3±0.1 | 12.6±2.4 | 6.2±1.7 | 0.03±0.01 | 0.002±0.001 |
| **Vitamin** | 14.4±2.2 | 7.8±1.8 | 0.2±0.1 | 0.002±0.001 | 9.1±1.8 | 57.0±7.2 | 2.6±0.2 | 13.0±2.6 | 3.1±1.1 | 0.01±0.004 | 0.002±0.001 |
| *p-value* | *1.000* | *1.000* | *1.000* | *1.000* | *1.000* | *0.329* | *1.000* | *1.000* | *0.220* | *0.514* | *1.000* |
| **LA** | 9.9±1.8 | 5.4±1.0 | 0.2±0.08 | 0.001±0.0004 | 7.2±1.7 | 65.5±4.6 | 2.4±0.3 | 10.9±1.3 | 3.0±1.2 | 0.01±0.004 | 0.001±0.0003 |
| *p-value* | *1.000* | *1.000* | *1.000* | *0.548* | *1.000* | *1.000* | *1.000* | *1.000* | *0.191* | *0.177* | *1.000* |
| **ALA** | 8.6±2.4 | 4.5±0.9 | 0.1±0.03 | 0.001±0.0004 | 6.1±1.3 | 59.7±9.4 | 2.7±0.5 | 11.5±1.5 | 1.9±0.8 | 0.01±0.004 | 0.0005±0.0002 |
| *p-value* | *0.910* | *0.744* | *1.000* | *0.686* | *1.000* | *0.737* | *1.000* | *1.000* | *0.028** | *0.077* | *1.000* |
| **AA** | 15.1±3.7 | 5.4±0.6 | 0.2±0.1 | 0.001±0.0004 | 8.3±1.1 | 63.2±6.3 | 2.5±0.2 | 14.4±1.6 | 0.2±0.07 | 0.002±0.0001 | 0.002±0.002 |
| *p-value* | *1.000* | *1.000* | *1.000* | *0.939* | *1.000* | *1.000* | *1.000* | *1.000* | *0.001**** | *0.004*** | *1.000* |
| **EPA** | 21.4±1.1 | 5.6±1.0 | 0.3±0.1 | 0.001±0.0004 | 9.1±1.9 | 58.7±4.9 | 3.3±0.3 | 16.7±2.2 | 0.4±0.2 | 0.006±0.003 | 0.003±0.002 |
| *p-value* | *0.296* | *1.000* | *1.000* | *0.555* | *1.000* | *0.395* | *0.100* | *0.871* | *0.002*** | *0.008*** | *1.000* |
| **DHA** | 14.3±2.7 | 5.3±0.7 | 0.2±0.08 | 0.001±0.0003 | 8.8±1.8 | 58.8±5.9 | 2.4±0.2 | 13.1±2.2 | 0.9±0.5 | 0.002±0.001 | 0.001±0.0008 |
| *p-value* | *1.000* | *1.000* | *1.000* | *0.489* | *1.000* | *0.630* | *1.000* | *1.000* | *0.004*** | *0.007*** | *1.000* |

**Supplementary Table 2:** Ratios of IFNγ, IL-13 and IL-10. Ratios were calculated by normalizing the data by dividing, for each individual donor, the production of either IL-13, IFNγ or IL-10 of each treatment by the production of the untreated DC2. The production of the DC2s for each cytokine were set to 1. Then, IL-13/IFNγ, IL-13/IL-10 and IFNγ/IL-10 ratios were calculated by dividing the means of IL-13 by the means of IFNγ, the means of IL-13 by the means of IL-10 and the means of IFNγ by the means of IL-10, respectively. Ratios are expressed in arbitrary units (AU). Means ± SEM. N=5 individual donors.
*** p<0.001

|  | **IL-13/IFNγ ratio** | **IL-13/IL-10 ratio** | **IFNγ/IL-10 ratio** |
| --- | --- | --- | --- |
| **NT** | 1 | 1 | 1 |
| **Vitamin** | 1.7±0.3 | 0.9±0.1 | 0.6±0.09 |
| *p-value* | *0.514* | *1.000* | *0.000**** |
| **LA** | 2.7±0.7 | 0.9±0.1 | 0.4±0.08 |
| *p-value* | *0.003**** | *1.000* | *0.000**** |
| **ALA** | 2.0±0.6 | 0.7±0.1 | 0.5±0.09 |
| *p-value* | *0.575* | *0.175* | *0.000**** |
| **AA** | 0.9±0.2 | 0.3±0.07 | 0.3±0.06 |
| *p-value* | *1.000* | *0.000**** | *0.000**** |
| **EPA** | 1.7±0.5 | 0.4±0.09 | 0.3±0.03 |
| *p-value* | *1.000* | *0.000**** | *0.000**** |
| **DHA** | 1.1±0.3 | 0.3±0.05 | 0.4±0.08 |
| *p-value* | *1.000* | *0.000**** | *0.000**** |
